# Factors associated with mucosal involvement in tegumentary leishmaniasis: a nation-based study using surveillance data from Brazil

**DOI:** 10.1590/S1678-9946202365047

**Published:** 2023-09-08

**Authors:** Clarisse Fonseca Monachesi, Adriano Gomes-Silva, Filipe Anibal Carvalho-Costa

**Affiliations:** 1 Fundação Oswaldo Cruz, Instituto Oswaldo Cruz, Laboratório de Epidemiologia e Sistemática Molecular, Rio de Janeiro, Rio de Janeiro, Brazil; 2 Fundação Oswaldo Cruz, Instituto Nacional de Infectologia Evandro Chagas, Laboratório de Pesquisa Clínica em Micobacterioses, Rio de Janeiro, Rio de Janeiro, Brazil; 3 Fundação Oswaldo Cruz, Instituto Oswaldo Cruz, Laboratório Interdisciplinar de Pesquisas Médicas, Rio de Janeiro, Rio de Janeiro, Brazil

**Keywords:** Cutaneous leishmaniasis, Mucosal leishmaniasis, Brazil

## Abstract

This study aimed to assess the factors associated with mucosal leishmaniasis (ML) within the scope of tegumentary leishmaniasis (TL) cases reported in Brazil. Surveillance data were assessed, and comparisons were made between ML and cutaneous leishmaniasis (CL) cases. Additionally, ML incidence rates for municipalities were depicted through a geographic information system. From 2007 to 2017, 235,489 TL cases were reported, of which 235,232 were classified as follows: 14,204 (6%) were ML cases and 221,028 (94%) were CL cases. Multivariate analysis showed that the proportion of ML cases reached 16.8% among individuals >75 years (adjusted OR = 2.77; 95% CI = 2.41-3.19; p < 0.001), and ML was also more frequent among males (aOR = 1.28; 95% CI = 1.20-1.38; p < 0.001), HIV-positive patients (aOR = 2.15; 95% CI = 1.80-2.56; p < 0.001), patients residing in urban areas (aOR = 1.52; 95% CI = 1.43-1.62; p < 0.001), and imported cases (with respect to county) when compared to autochthonous cases (aOR = 1.84; 95% CI = 1.71-1.98; p < 0.001). A lower proportion of positive results in direct parasitological examinations was observed in ML cases (32.6% vs. 60.8%; p < 0.001). The leishmanin skin test results were more often positive in ML cases (41.7% vs. 25.9%; p < 0.001). In ML, compatible changes in histopathology were more frequent (14.6% vs. 3.9%; p < 0.001). A greater proportion of ML cases were treated with amphotericin B (6.9% vs. 0.9%; p < 0.001). The case-fatality rate was higher in ML (0.6% vs. 0.1%; p < 0.001). A higher incidence of ML was observed in a geographical band extending across the Amazon region from the southern Para State to the Acre State. ML exhibited varying frequencies within specific populations. The definition of predictable factors predisposing *Leishmania*-infected subjects to develop ML is important for defining strategies to mitigate the mucosal damage caused by leishmaniasis.

## INTRODUCTION

Leishmaniases, including tegumentary leishmaniasis (TL) and visceral leishmaniasis (VL), are vector-borne neglected parasitic diseases considered major public health problems in many regions^1^. The World Health Organization estimates that 350 million people are at risk, with an estimated 2 million new cases of TL and VL reported annually^1^.

In Brazil, seven *Leishmania* species have been identified as causative agents of TL: *Leishmania (Viannia) braziliensis, L. (V.) guyanensis, L. (Leishmania) amazonensis, L. (V.) lainsoni, L. (V.) naiffi, L. (V.) lindenberg* and *L. (V.) shawi*. Vectors of leishmaniases are phlebotomines (sandflies), insects belonging to the order Diptera and family Psychodidae. The main sandfly species involved in TL transmission in Brazil are *Lutzomyia flaviscutellata, Lu. whitmani, Lu. umbratilis, Lu. intermedia, Lu. wellcomei and Lu. migonei.* In the New World, infections are predominantly zoonotic and have been described in various species of wild animals, such as rodents, marsupials, edentates, chiroptera, and wild canids, as well as in domestic animals such as dogs, cats and horses^2^.

Leishmaniases manifest across a wide clinical spectrum and TL can be classified into two main clinical forms: cutaneous leishmaniasis (CL) and mucosal leishmaniasis (ML). This dichotomous classification can be considered a simplification since a variable proportion of TL cases classified as CL present unnoticed oral and nasal mucosal lesions.

ML is a potentially mutilating clinical phenotype and, therefore, is a severe variation characterized by destructive lesions on the mucous membranes of the upper respiratory tract (inner nostril wall, oral cavity, pharynx, and larynx)^3^. *L. (V.) brasiliensis* is the main cause of ML in the American continent, but the disease is also caused by *L. (L.) amazonensis, L. (V.) panamensis*, and *L. (V.) guyanensis* ^4-6^. Despite being more commonly reported in the American continent, attention has also been paid to its occurrence in the Old World. In contrast to the predominantly nasal presentation in the New World, Old World ML usually affects the oral cavity, pharynx and larynx in patients infected with *L. donovani*, frequently associated with VL^7^. Mucosal lesions caused by *L. tropica* and *L. major* have been reported in the Middle East^8^.

ML can be classified into different forms: i) late (the most common form, representing the appearance of mucosal lesions after long-term CL lesions that have healed), ii) with no previous CL lesions (possibly associated with small CL lesions or non-ulcerated and/or unnoticed CL lesions), iii) concomitant with CL lesions (less common and associated with immunodeficiency), iv) contiguous with CL lesions (by direct spread of skin lesions located near natural orifices), and v) primary (by direct inoculation of the parasite in the mucosa)^2^.

The proportion of ML cases in the universe of TL cases shows great variation across different regions and populations and, in Brazil, tends to be more prevalent in areas with lower TL endemicity^9^. The factors associated with mucosal involvement in leishmaniasis are not fully understood and probably involve a network of interconnected determinants represented by host immune responses, socioecological factors and parasite genotypes. It has been proposed that *Leishmania* strains genetically adapted to the mucosal epithelium of the upper respiratory tract, and the presence of *Leishmania* RNA viruses (LRV) could drive pathogenesis towards mucosal involvement^10-13^. Substances with a potential immune regulatory role in sandfly saliva could also influence the clinical outcomes of TL^14^. The host immune response is an important factor, and ML is a hyperergic and pauciparasitic condition characterized by an intense cellular anti-*Leishmania* response and a scarcity of parasites, leading to strongly positive leishmanin skin tests^15^. Interferon-𝛾 and tumor necrosis factor-𝛼 production is higher in ML, whereby CD4+ T cells are the major source of cytokines; this exacerbated Th1 immune response promotes tissue destruction^15^. It has been proposed that patients with cellular immunity deficiency, including HIV-positive patients, lose the capacity to modulate the inflammatory response and become more likely to develop ML^16^. ML is also associated with old age, chronicity, delayed diagnosis and a lack of access/adherence to treatment^17^. In Brazil and in other endemic countries, the factors associated with ML are not well described. In this study, we assess the clinico-epidemiological factors associated with mucosal involvement in the universe of TL cases reported in Brazil from 2007 to 2017.

## MATERIAL AND METHODS

### Description of the study area

In 2021, Brazil had an estimated population of 213,317,639 inhabitants^18^ spread over 8,515,767 km^2^, which corresponds to 48% of the total area of South America. Brazil is divided into five main geographical regions: the North (population=18,906,962 inhabitants), the Northeast (57,667,842), the Central-West (16,707,336), the Southeast (89,632,912) and the South (30,402,587)^18^. Brazil has 26 federative units (states) and one Federal District, displaying enormous socioecological diversity, its population has undergone a process of increasing urbanization. The 5,570 Brazilian municipalities (counties) have human development indexes ranging from 0.418 (very low) to 0.882 (very high) and are situated in a wide variety of demographic and environmental scenarios. Among recent changes, there has been great expansion of mechanized agriculture for staple food production and livestock pastures, associated with deforestation. Brazilian physiogeography includes different biomes and climates: the Amazon (North), the semi-arid Caatinga (Northeast), the Cerrado (Central Brazil), the Atlantic Forest (Coastal Strip) and the Pampas (South). TL is prevalent in all Brazilian biogeographic regions, each characterized by distinct incidence rates.

### Data source for tegumentary leishmaniasis in Brazil and case definition

Information regarding TL in Brazil was assessed through surveillance data generated from case notification forms from 2007 to 2017. TL is a mandatory notifiable disease in Brazil and its treatment requires formal notification. Cases are reported through standardized forms submitted to the Information System for Notifiable Diseases (SINAN, Sistema Nacional de Agravos de Notificacao) of the Brazilian Ministry of Health (BMoH). TL notification forms encompass the following variables: gender, age, clinical form (CL or ML), county, state, co-infection status with HIV, results of diagnostic tests (direct observation of the parasite in dermal scrapings, leishmanin skin test, histopathology), drugs used for the treatment, criteria for confirmation and clinical outcomes. According to BMoH guidelines, CL is defined by the presence of single or multiple cutaneous ulcers with granular bases and infiltrated margins, confirmed either through laboratory evidence or clinico-epidemiological criteria.; ML is defined by the presence of one or more ulcers in the nasal mucosa, with or without perforation or loss of the nasal septum, and the possibility of involvement of the lips and mouth (palate and nasopharynx). TL treatment is freely provided by the Unified Health System, comprising a 20-day regimen of N-methyl-glucamine antimoniate for CL and a 30-day regimen for ML. When this treatment fails, amphotericin B is used. For regions where *L. guyanensis* predominates, the use of pentamidine isethionate is preferably recommended as the first-choice drug. More recently, treatment of CL with miltefosine orally, on a 28-day basis, was implemented in Brazil. The success of the treatment is defined as the epithelialization of the ulcerated lesions and the total regression of the infiltration and erythema, up to three months after the completion of the therapeutic regimen. TL cases are reported to BMoH by primary health centers, outpatient clinics and hospitals.

### Study design and strategy of analysis

**Figure 1:**
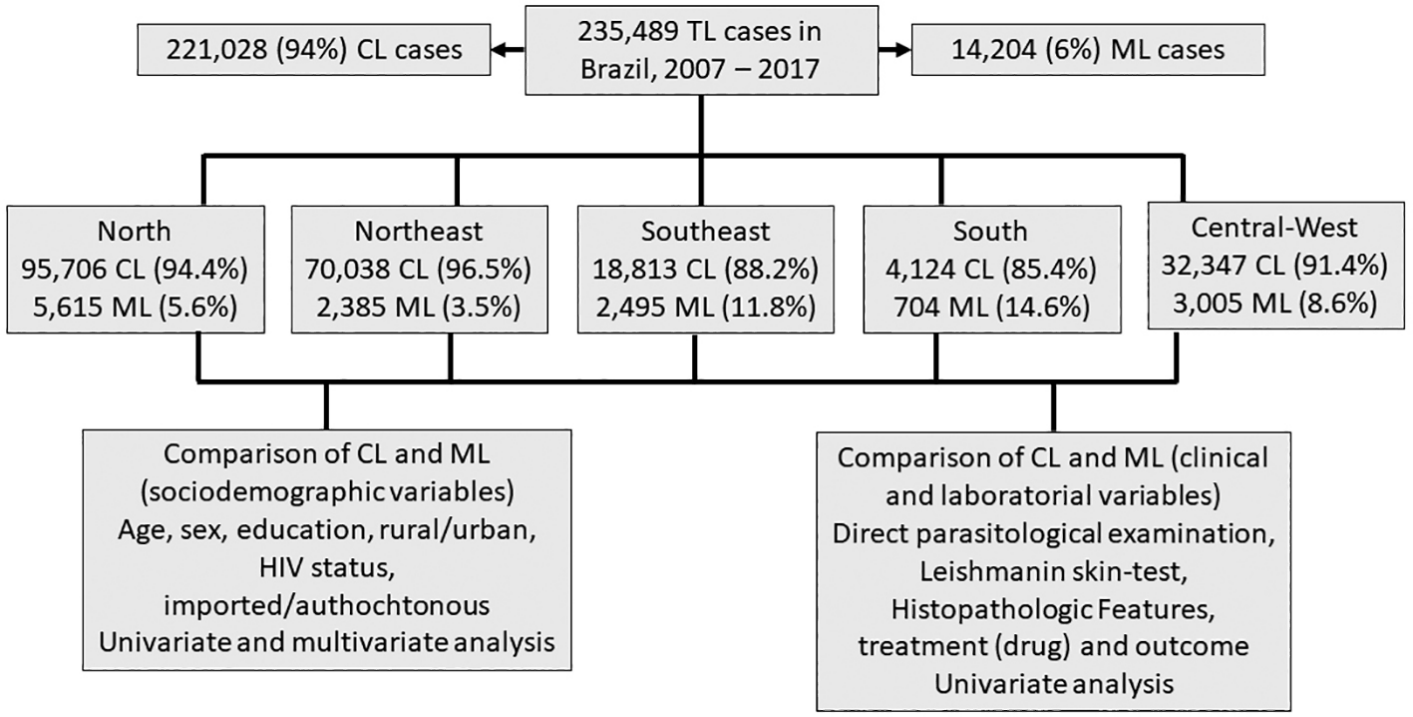
Study flowchart showing the number of patients per region and the proportion of different clinical forms (cutaneous leishmaniasis and mucosal leishmaniasis).

In a case-comparison study, patients with ML and CL were compared with respect to age, gender, housing area (urban or rural), presence of HIV coinfection, level of schooling and whether the case was autochthonous or imported in relation to the reporting municipality (Figure 1). This binary classification, adopted by BMoH, was used for the purposes of analysis and calculation of association measures, under the recognition that the clinical spectrum of TL is more complex, which may involve overlapping clinical forms in some cases, including mucosal lesions not diagnosed by inaccurate physical examination. Univariate and multivariate analyses (logistic regression) were performed and crude and adjusted odds ratios (ORs) were calculated; in the univariate model, the statistical significance of the associations was assessed using the chi-square test. In addition, clinical, laboratory, and treatment data were analyzed, comparing ML and CL cases including, as independent variables, the results of direct parasitological examination, leishmanian skin testing and histopathological examination, the drug used in the treatment (pentavalent antimonial, amphotericin B or pentamidine), and clinical evolution (cure, abandonment of treatment, or death) **(** Figure 1 **)**. EpiInfo 2000 software (version 3.5.1, CDC, Atlanta, GA, USA) was used for all calculations. Cases with change in diagnosis were excluded from the analyses. Annual ML incidence rates were calculated as the number of new ML cases in a year / population x 100,000. For these calculations, only cases with mucosal involvement were used in the numerator, thus excluding cases of CL. The rates were calculated for regions and municipalities using official annual population estimates from 2007 to 2017. The average annual rates for municipalities were used to build a map using QGIS software (QGIS Development Team) to verify the areas where ML occurs most frequently in Brazil.

### Ethical approval

The study was not required to obtain ethical clearance as it was conducted using free-access secondary data.

## RESULTS

### Factors associated with mucosal involvement in tegumentary leishmaniasis

From 2007 to 2017, a total of 235,489 TL cases were reported, of which 235,232 (99.9%) cases were classified as follows: 221,028 (94%) were CL and 14,204 (6%) were ML (Figure 1). As presented in Table 1, significantly lower rates of ML were observed in patients aged 0-15 years and 16-30 years. Older age groups exhibited up to three-fold higher rates of mucosal involvement, reaching 16.8% among individuals over 75 years old. Mucosal involvement was significantly more prevalent in male patients. Among the reported TL cases in Brazil during this period, 115,272 (49%) had the HIV status described in the notification form, with 2,050 (1.8%) being HIV-positive; these patients were found to have a mucosal involvement rate twice as high as that of HIV-negative patients. Patients residing in urban areas were more often classified as ML at the time of notification compared to patients residing in rural areas. A significantly higher rate of mucosal involvement was also observed in cases considered imported in relation to the municipality of notification when compared to autochthonous cases.

**Table 1:** Uni and multivariate analysis to assess factors associated with the mucosal form of tegumentary leishmaniasis in Brazil, including all cases reported from 2007 to 2017.

	Proportion with mucosal involvement	Crude Odds ratio (95% confidence interval)	p-value	Adjusted Odds ratio (95% confidence interval)	p-value
Age group					
0 – 15 years	1,233/38,208 (3.2%)	0.53 (0.50-0.57)	<0.001	0.50 (0.44-0.58)	<0.001
16 – 30 years	2,647/75,484 (3.5%)	0.58 (0.55-0.61)	<0.001	0.58 (0.53-0.63)	<0.001
31 – 45 years	3,408/58,158 (5.9%)	1		1	
46 – 60 years	3,396/37,073 (9.2%)	1.62 (1.54-1.70)	<0.001	1.63 (1.50-1.77)	<0.001
61 – 75 years	2,494/17,656 (14.1%)	2.64 (2.50-2.79)	<0.001	2.66 (2.43-2.92)	<0.001
≥ 76 years	868/5,177 (16.8%)	3.23 (2.98-3.50)	<0.001	2.77 (2.41-3.19)	<0.001
Sex					
Male	10,854/170,966 (6.3%)	1.23 (1.18-1.28)	<0.001	1.28 (1.20-1.38)	<0.001
Female	3,347/64,242 (5.2%)	1		1	
Education level					
No education or basic level	5,946/85,201 (7%)	1.40 (1.35-1.46)	<0.001	1.06 (0.99-1.13)	0.064
Intermediate level	4,160/82,403 (5.1%)	1		1	
High school or university	261/4,601 (5.7%)	1.12 (0.99-1.28)	0.052	0.78 (0.65-0.93)	0.006
Residential area					
Urban	7,975/99,439 (8%)	1.86 (1.80-1.93)	<0.001	1.52 (1.43-1.62)	<0.001
Rural	5,665/127,086 (4.5%)	1		1	
HIV status					
Positive	288/2,050 (14%)	2.24 (1.98-2.55)	<0.001	2.15 (1.80-2.56)	<0.001
Negative	7,676/113,223 (6.8%)	1		1	
Epidemiological classification					
Imported	2,279/25,340 (9%)	1.82 (1.73-1.91)	<0.001	1.84 (1.71-1.98)	<0.001
Autochthonous	9,940/193,282 (5.1%)	1		1	

### Clinical, laboratory, and treatment data in cutaneous and mucosal leishmaniasis

As presented in Table 2, a decreased proportion of positive outcomes in direct parasitological examinations was observed in patients with ML when compared to CL cases. The leishmanin skin test was significantly more often positive in ML. The presence of amastigote parasites in the histopathologic examination was similar in both groups. In ML, compatible changes in histopathology were more frequent than in CL. A substantially greater proportion of ML cases was treated with amphotericin B. The case-fatality rate was six times higher in ML when compared to CL.

**Table 2:** Comparison of cases of mucosal leishmaniasis and cutaneous leishmaniasis in relation to laboratory, therapeutic and outcome aspects, including all cases reported in the period from 2007 to 2017.

	Mucosal leishmaniasis (n=14,204)	Cutaneous leishmaniasis (n=221,028)	p-value
Direct parasitological examination			
Positive	4,632 (32.6%)	134430 (60.8%)	<0.001
Negative	1,301 (9.3%)	12913 (5.8%)	
Not performed	8,271 (58.2%)	73685 (33.3%)	
Leishmanin skin-test			
Positive	5,930 (41.7%)	57214 (25.9%)	<0.001
Negative	698 (4.9%)	8193 (3.7%)	
Not performed	7,576 (53.3%)	155621 (70.4%)	
Histopathologic Features			
Positive for TL^a^	1,591 (11.2%)	22521 (10.2%)	
Compatible with TL^b^	2,078 (14.6%)	8551 (3.9%)	<0.001
Not compatible with TL	739 (5.2%)	6786 (3.1%)	
Not performed	9,796 (69%)	183170 (82.9%)	
Drug for treatment			
N-methyl-glucamine antimoniate	11,138 (83.8%)	197087 (93.2%)	
Amphotericin B	916 (6.9%)	1921 (0.9%)	<0.001
Pentamidine	181 (1.4%)	2732 (1.3%)	
Other drug	748 (5.6%)	7216 (3.4%)	
Not treated	311 (2.3%)	2480 (1.2%)	
Outcome			
Recovery	9,475 (87.2%)	160200 (93.6%)	
Non-adherence to treatment	411 (3.8%)	5642 (3.3%)	
Death due to TL	67 (0.6%)	128 (0.1%)	<0.001
Death not related to TL	256 (2.4%)	848 (0.5%)	
Health unity transfer	364 (3.3%)	2085 (1.2%)	
Change of diagnosis	293 (2.7%)	2255 (1.3%)	

^a^Presence of amastigotes; ^b^Tuberculoid granulomas with associated lymphoplasmacytic inflammatory infiltrate and, occasionally, necrosis.

### Proportion of cases with mucosal involvement and incidence rates of mucosal leishmaniasis in distinct Brazilian regions

**Figure 2:**
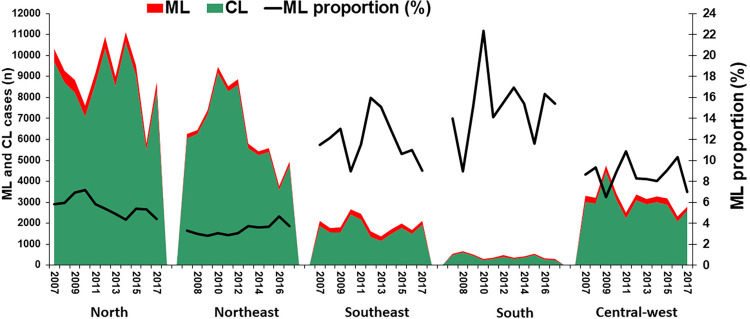
Number of cases of cutaneous and mucosal leishmaniasis and proportion of cases with mucosal involvement by year and region in Brazil, from 2007 to 2017.

**Figure 3:**
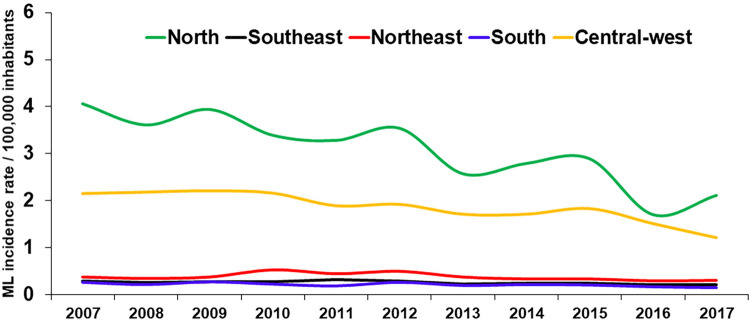
Annual incidence rates of mucosal leishmaniasis by Brazilian region, 2007 to 2017.

**Figure 4:**
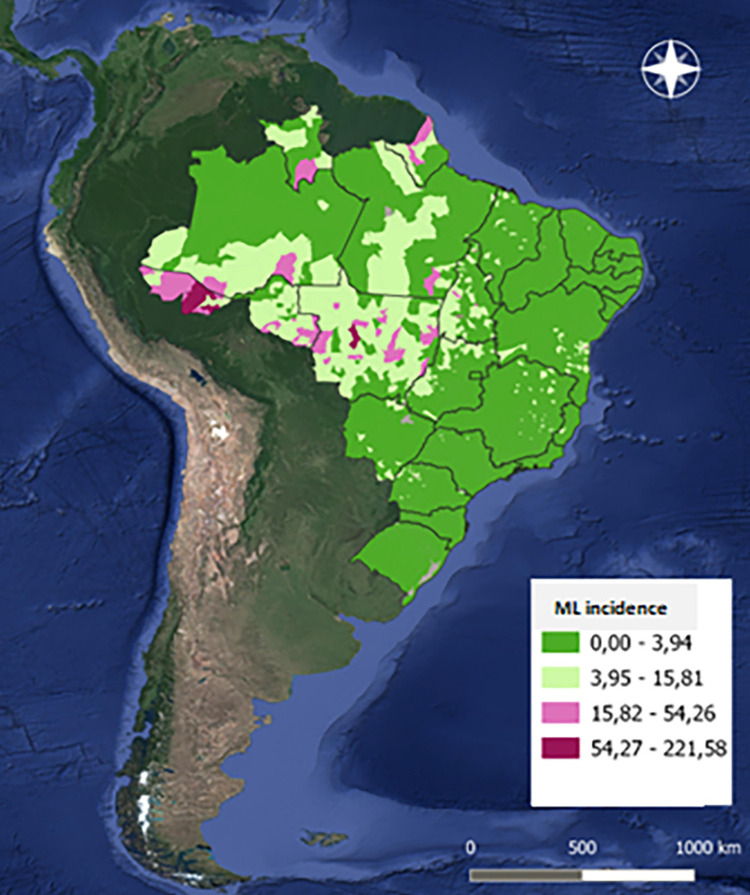
Map detailing the average annual incidence rates between 2007 and 2017, on a municipal basis, made with the QGIS program.

The proportion of TL cases that were classified as ML between 2007 and 2017 across different Brazilian regions is presented in Figure 2. The North and Northeast regions registered the highest number of CL and ML cases, followed by the Central-West. The highest proportions of cases classified as ML, which ranged from 8.9% to 22.4%, were observed in the regions with the lowest number of cases, namely the South and the Southeast. In the North, the proportion of cases classified as ML ranged from 4% to 7.2%. Figure 3 shows the annual incidence rates of ML in the different Brazilian regions, demonstrating the heterogeneity of the disease distribution, which occurs much more frequently in the North, followed by the Central-West. The map in Figure 4 offers more details on the spatial distribution of ML, showing a territorial band with the highest incidence extending from the southern Para State to the Acre State, both in the Amazon region.

## DISCUSSION

In this study, we assessed the factors associated with mucosal involvement among patients with TL in Brazil. The study demonstrated that ML is significantly more common in older individuals, with a frequency that increases substantially with advancing age. The low proportion of ML among young patients is well described in hospital- and field-based case series in Brazil and Bolivia^3,17,19^. The recurrence of the disease, affecting the mucosa during old age after the healing of cutaneous ulcers associated with CL, suggests that the immunosenescence phenomenon may be involved in this clinical phenotype^20^.

ML immunopathogenesis is based on the exacerbation of the cellular immune response, associated with the inability to modulate the proinflammatory signals^21,22^. It has been proposed that immunosenescence is of particular relevance in diseases caused by intracellular pathogens and could disturb the immunoregulation capacity, allowing the appearance of tissue damage caused by the inability to prevent the exacerbation of the immune system effector functions, producing a more severe clinical presentation. Nevertheless, this is a topic that needs to be addressed in more depth in clinical and experimental studies. An active TL surveillance in Bolivia revealed a significant increase in the rate of mucosal involvement parallel with age, demonstrating a proportion of ML of 36% in patients over 65 years of age^17^.

Factors linked to disease chronicity may also contribute to the greater proportion of ML in the elderly. Among these factors, the lack of access to diagnosis and treatment during the early stages of the disease in remote areas is significant. Especially among people living in the Amazon region, where *L. (V.) guyanensis* is more frequently associated with TL, treatment with N-methyl-glucamine antimoniate can present a significant failure rate^23^. This can contribute to *Leishmania* infection chronicity and increase the risk of it evolving into ML. The demonstration of *Leishmania* DNA in intact and unaffected mucosa of patients with TL also helps to clarify the role of the parasite’s persistence and the development of mucosal lesions^24^.

We show that the proportion of mucosal involvement among HIV-positive patients is more than twice that of HIV-negative individuals. Thus, although ML is a hyperergic state with a low parasite load, the dysregulation of the immune response that occurs in the course of an HIV infection increases the likelihood of mucosal involvement. This can be explained by the dysfunction of the T and B regulatory lymphocytes responsible for the anti-inflammatory roles that impair the immune response hyperactivation^25^. In French Guiana, a case-control study with a small number of patients with TL found ML only in HIV-positive patients, who also had higher rates of recurrence and reinfection^26^. In a case series, a higher proportion of mucosal lesions was also observed in HIV-positive patients with TL in Brazil^27^. Considering Old World leishmaniasis caused by parasites belonging to the *L. donovani* complex, the association of HIV infection and ML has been demonstrated in Italy and Spain^28,29^.

Another factor potentially associated with an increased risk of evolving into ML is the presence of LRV, which is thought to activate a higher inflammatory immune response by innate immune system receptors^30^. An association between the presence of LRV and the occurrence of therapeutic failure in TL was observed in the Amazon region^31-33^. Nevertheless, the correlations of LRV with distinct TL clinical phenotypes, treatment failure, and disease severity require deeper clinical and laboratory investigations.

The proportion of patients affected with the mucosal form of the disease was approximately 25% higher among males, a finding that can suggest lower demand for health services and less adherence to treatment among men. Lower adherence to the treatment for TL has been reported among men, as well as lower cure rates; however, sex differences in ML rates may also be related to the host’s immunological status and the adaptive immune responses of both sexes^34^. It is important to note that ¾ of the patients with TL in Brazil from 2007 to 2017 were males, which demonstrates that, in addition to a higher rate of ML, men also have a higher incidence of TL, regardless of the clinical form. This is probably caused by the greater male exposure associated with work, including plant extractivism, agriculture, mining and livestock activities^35^. A recent review also observed a significantly higher incidence of ML in male patients in South America^19^. Whether the greater burden of ML in men is a phenomenon associated with occupational risk or whether there are genetic differences in the immune response of males and females is still a matter of debate.

The multivariate model showed that having tertiary education confers protection against the development of ML, evidencing the higher vulnerability of less educated patients, who probably belong to the poorest social strata and have the worst access to diagnosis and poor adhesion to treatment. The proportion of TL cases with mucosal lesions was almost twice as high in patients living in urban areas than in those living in rural areas. This may be associated with migration from rural areas to the cities and suggests disease chronicity. In this way, the diagnosis of ML is made at a late stage when the patient has already left the most active transmission zones. This is probably also the reason why a higher proportion of ML is observed in imported cases, compared to autochthonous cases (in relation to the municipality). Access to more accurate clinical screening for the detection of mucosal lesions in patients with TL could also be influencing the higher proportion of subjects with ML in urban regions. The notification of TL in Brazil classifies the case as autochthonous or imported in relation to the municipality, and the municipality in which the patient probably contracted TL is also investigated. However, the latter information has low accuracy, especially in cases of ML, when the initial infection normally occurred many years ago.

ML case series from the Amazon region showed that the vast majority of patients presented skin lesions in the past, whereby the mean time elapsed between the skin lesion and the appearance of ML was 16.3 years^36^. A case series of TL cases imported from South America to Israel demonstrated that the absence of previous systemic antiparasitic treatment is a significant risk factor for developing ML in the evolution of TL^37^. The drug N-methyl-glucamine antimoniate is associated with lower rates of adherence to antiparasitic treatment, which theoretically predisposes patients to chronic infection^38^.

The comparison of ML and CL cases with respect to laboratory data demonstrates the differences in pathophysiology and the need for different diagnostic approaches. Positivity to direct parasitological examination was less frequent in cases of ML, with reactivity to leishmanin skin testing being more frequent in this form. It is noted that a greater proportion of cases of ML required treatment with the second-line drug, amphotericin B. Although the TL case-fatality rate is low, it is six times higher than in ML, denoting its potential to cause life-threatening injuries to the upper respiratory tract.

We showed that the Amazonian population is the most affected by ML in Brazil, an aspect already well demonstrated and known^2^. The incidence rates of municipalities (counties) reveal a territorial strip of higher risk, which reasonably corresponds to the south of the Amazon, extending from the southern Para State and the Tocantins State towards the Western Amazon in the Acre State, passing through Mato Grosso State and Rondonia State. This belt is spatially correlated with the so-called arc of deforestation, a zone of expansion of the agricultural frontier that has great potential for TL transmission. A study carried out in the Purus River basin, located in this strip, showed a high proportion of TL cases classified as ML (20.8%), which is probably related to the difficulty in accessing diagnosis and treatment^39^. The association between leishmaniasis and socioecological aspects has been demonstrated in the Brazilian Amazon^40^. These data strengthen the view that one of the determinants of ML is the quality of care and the ability of the primary health care system to detect early evolving cases. In rural Bolivia, a project with active TL surveillance and improved access to treatment carried out by non-governmental organizations was able to reduce the proportion of mucosal involvement from 25% to 2% over six years^22^.

ML may be more frequent in the initial presentation of TL, but small nasal lesions are likely to go unnoticed since many are incipient and require a more accurate rhinoscopy. These lesions would progress with treatment failure, leading to cases of ML. Commonly used drugs may be suboptimal for parasitological treatment and, even if the skin lesions heal, the elimination of the parasite is not guaranteed. The persistence of *Leishmania* keeps the immune system in constant activation and, in the presence of immune disorders, including aging and HIV infection, the patient loses the ability to respond in a modulated way, leading to mucosal tissue damage.

A limitation of the present study is the adoption of a dichotomous clinical classification, based on a variable from the notification form, which in a certain proportion of cases may be imprecise since there is a potential overlap in the spectrum of disease presentation. This overlap occurs due to the presence of mucosal lesions in cases that are classified as CL, which could theoretically reduce the accuracy of our data. However, we chose to follow the classification of the notification form as this allowed the calculation of measures of association and the description of factors associated with mucosal involvement in TL.

## CONCLUSION

In conclusion, ML is a clinical phenotype more frequent in specific populations. Policies to reduce the occurrence of severe cases of TL could include improved early diagnosis and the recognition of more vulnerable populations in high-risk areas. The definition of predictable factors predisposing *Leishmania*-infected subjects to develop ML is of paramount importance in defining strategies to avoid the mucosal damage caused by leishmaniasis.
